# What Do Parents of Children with Down Syndrome Think about Non-Invasive Prenatal Testing (NIPT)?

**DOI:** 10.1007/s10897-016-0012-4

**Published:** 2016-09-13

**Authors:** Rachèl V. van Schendel, Adriana Kater-Kuipers, Elsbeth H. van Vliet-Lachotzki, Wybo J. Dondorp, Martina C. Cornel, Lidewij Henneman

**Affiliations:** 1 Department of Clinical Genetics, Section of Community Genetics, EMGO Institute for Health and Care Research, VU University Medical Center, Amsterdam, The Netherlands; 2 Dutch Genetic Alliance (VSOP), Soest, The Netherlands; 3 Department of Health, Ethics and Society, Faculty of Health, Medicine and Life Sciences, Research Institutes GROW and CAPHRI, Maastricht University, Maastricht, The Netherlands

**Keywords:** Qualitative research, Prenatal diagnosis, Prenatal screening, Non-invasive prenatal testing, NIPT, cfDNA, Down syndrome, Attitude, Parents, Counseling

## Abstract

This study explores the attitudes of parents of children with Down syndrome towards non-invasive prenatal testing (NIPT) and widening the scope of prenatal screening. Three focus groups (*n* = 16) and eleven individual interviews with Dutch parents (and two relatives) of children with Down syndrome were conducted. Safety, accuracy and earlier testing were seen as the advantages of NIPT. Some participants were critical about the practice of screening for Down syndrome, but acknowledged that NIPT enables people to know whether the fetus is affected and to prepare without risking miscarriage. Many feared uncritical use of NIPT and more abortions for Down syndrome. Concerns included the consequences for the acceptance of and facilities for children with Down syndrome, resulting in more people deciding to screen. Participants stressed the importance of good counseling and balanced, accurate information about Down syndrome. Testing for more disorders might divert the focus away from Down syndrome, but participants worried about “where to draw the line”. They also feared a loss of diversity in society. Findings show that, while parents acknowledge that NIPT offers a better and safer option to know whether the fetus is affected, they also have concerns about NIPT’s impact on the acceptance and care of children with Down syndrome.

## Introduction

Non-invasive prenatal testing (NIPT) using cell-free placental DNA is increasingly being used to test for fetal aneuploidy. By using a maternal blood sample, NIPT can test for Down syndrome with a sensitivity of more than 99 % and a false-positive rate of less than 0.1 % (Gil et al. 2015). For women with an elevated risk based on the first-trimester combined test (FCT), NIPT is a safe alternative to invasive testing, although invasive testing will be required to confirm a positive NIPT result. Due to its high accuracy, NIPT can also be used as a first-tier screening test for all pregnant women, thereby replacing the FCT (Benn et al. 2015), although the positive predictive value is significantly lower in lower-risk women as compared to high-risk women (Norton et al. 2015). The introduction of this innovative test is having great impact on the prenatal landscape. Furthermore, it has been proven possible to scan the whole fetal genome with NIPT (Lo et al. 2010), so future use is likely to expand to testing for a wider range of genetic disorders.

Several studies have investigated the attitudes towards NIPT of important stakeholders such as health professionals and pregnant women. Overall, these studies show that both pregnant women (Farrell et al. 2014; Lewis et al. 2013; van Schendel et al. 2014) and health professionals (Musci et al. 2013; Tamminga et al. 2015) have great interest in NIPT due to its ability to test early in pregnancy with high accuracy and no miscarriage risk. However, concerns were expressed about potential “routinized” or uncritical use of NIPT, women feeling pressure to test, and the possible impact of NIPT on acceptance of people with a disability (Lewis et al. 2013; van Schendel et al. 2014). Alongside these concerns, the introduction of NIPT in routine prenatal care has been criticized (Kaposy 2013), on the basis of the disability rights critique (Kaposy 2013; Parens and Asch 2003). It has been argued that prenatal screening for Down syndrome sends out a message that emphasizes the negative aspects of living with Down syndrome, and implementing NIPT runs counter to the hope of improving attitudes towards Down syndrome (Kaposy 2013).

Very little is known about what parents of children with Down syndrome think about prenatal screening and, in particular, about NIPT. Using an online survey, Kellogg et al. (2014) studied the attitudes of 73 US mothers of children with Down syndrome towards NIPT. They showed that the majority of mothers agreed that NIPT should be available to all pregnant women, and that NIPT was a good thing because it allows people to prepare themselves for a child with Down syndrome. However, most of the mothers also expected NIPT to cause an increase in pressure to test and in social stigma for having a child with Down syndrome (Kellogg et al. 2014). When looking at the attitudes of parents of children with Down syndrome towards prenatal testing in general, it seems that most believe prospective parents should have autonomy and reproductive freedom (Inglis et al. 2012; Scott et al. 2013). However, studies have shown cultural and religious differences in attitudes towards prenatal testing and termination of pregnancy amongst parents of children with Down syndrome (Ahmed et al. 2013; Bryant et al. 2011). A study of 78 women who had a sibling with Down syndrome showed that they overall had a positive experience of having a brother or sister with this condition, but around one-third would still consider prenatal testing and termination of pregnancy since they experienced a negative impact on themselves and their family (Bryant et al. 2005).

Decisions in a national screening system need political support, thus taking account of many perspectives. Since the introduction of NIPT could have an impact on the way society perceives Down syndrome and the lives of people living with this condition, it is important to further investigate what parents of children with Down syndrome think about introducing NIPT into a national prenatal screening system and which consequences they think this will have. This information can be used to establish a responsible implementation of NIPT, taking account of all stakeholder perspectives. This study therefore addresses the following research questions: 1) What do parents of children of Down syndrome think are the advantages and disadvantages of using NIPT for prenatal screening?; 2) What are important requirements for a responsible NIPT offer according to them?; and 3) What do they think about widening the scope of prenatal testing with NIPT?

This study was performed in the Netherlands, where the uptake of prenatal screening for Down syndrome (and trisomy 18 and 13) is relatively low (~27 %) (Schielen 2010) compared to nearby countries like Denmark (90 %) (Ekelund et al. 2011) or England (74 %) (National Health Service 2012). The low uptake of screening might be partially explained by the way screening is offered to women, with a clear emphasis on the “right not to know,” women having to pay for FCT (Crombag et al. 2014), and the rather positive attitudes towards Down syndrome in the Netherlands (Bakker et al. 2012; Crombag et al. 2016).

## Methods

A qualitative research design was used. Focus groups were formed to explore multiple perspectives and to stimulate discussion. Additional individual, semi-structured interviews were held to allow for a more private environment to explore the attitudes and (often emotional) experiences of parents of children with Down syndrome. Ethical approval for this study was obtained from the Medical Ethical Committee of the VU University Medical Center Amsterdam (VUMC). Informed consent was obtained from all individual participants included in the study.

### Participants

Participants were recruited with help of the Dutch Genetic Alliance (VSOP). An invitation for participation was placed on the website of the Dutch Down Syndrome Foundation (SDS, parent organization). As this produced no responses, another invitation was placed on a closed Facebook group consisting of about 900 members sharing experiences of having a child with Down syndrome. In total, 58 parents responded to the invitation, and two parents were recruited through the researchers’ network. A total of 27 people took part in the study; 16 participated in the three focus groups (each consisting of 5 to 6 participants) and 11 in an individual interview. The parents who participated in the focus groups were not related. Two of the focus group participants were not parents but relatives of a child with Down syndrome (sister and aunt). Participant characteristics are summarized in Table 1. After three focus groups and 11 interviews no new information was obtained, and therefore data saturation was reached.

**Table 1:** Characteristics of participants in the three focus groups and individual interviews

Characteristic	Focus groups (*n* = 16)	Individual interviews (*n* = 11)
Sex		
Female	14	9
Male	2	2
Mean age, years (range)	39.7 (29–50)	41.1 (31–48)
Level of education^a^		
Low	0	0
Medium	1	4
High	15	7
Religion		
None	12	9
Christian	4	2
Mean number of children (range)	2 (0–3)	2 (1–4)
Number of children with DS		
0	2^b^	0
1	13	10
2	1	1
Mean age of child with DS, years (range)	6 (1–17)	6 (1–16)
Prenatal screening during pregnancy of child with DS		
*Yes:*		
Low-risk FCT result	2	2
Low-risk FCT result, invasive test after ultrasound abnormality	1	0
High-risk FCT result, no invasive test	2	0
High-risk FCT result, invasive test	0	1
FCT (result unknown)	1	0
*No:*		
Not interested	5	7
Not offered	3	0
Declined screening because of the costs	0	1
*Not applicable:*	2^b^	0

*DS*=Down syndrome, *FCT* First-trimester combined test

^a^Low: elementary school, lower level of secondary school, lower vocational training; Medium: higher level of secondary school, intermediate vocational training; High: higher vocational training, university

^b^Two relatives of children with DS, a sister and an aunt

### Instrumentation and Procedures

In April 2014 NIPT became available in the Netherlands in public healthcare as a second-tier screening test. The first two focus groups were conducted prior to this period, in September 2013, in a community center in the middle of the Netherlands (Utrecht). The last focus group was in April 2015 at the VUMC in Amsterdam. The individual interviews were conducted by A.K.K. between March and April 2015, and took place at participants’ home, workplace or by telephone.

The focus group sessions were conducted using a semi-structured interview guide based on the one used in our previous study of pregnant women and their partners (van Schendel et al. 2014). The guide included the following topics: participants’ perceptions of the current Down syndrome screening using the FCT and invasive tests; perceptions of the advantages and disadvantages of NIPT, especially when NIPT would become available as a first-tier screening test; and opinions about testing for a wider range of disorders using NIPT. Via a PowerPoint presentation, participants were given a brief explanation of the characteristics of the current screening program and characteristics of NIPT, including testing for more genetic disorders. The focus groups were managed by an experienced moderator, together with an assistant taking notes and observing group interactions. For the individual interviews, the same semi-structured interview protocol, with some minor changes, was used. During the individual interviews, information about the current screening program and NIPT was provided verbally, supported by illustrations.

### Data Analysis

Focus groups and interviews were audiotaped and transcribed verbatim. After transcription, a thematic content analysis was performed using the qualitative software program ATLAS.ti 5.2. Responses in the text were coded independently by R.v.S. and A.K.K., and ranked and clustered into main topics and subtopics in order to identify important themes. Themes and codes were discussed with a third researcher (L.H.), and discrepancies were discussed until consensus was reached. Representative quotes from the focus groups (FG) and interviews (I) were translated from Dutch and are presented to illustrate the themes.

## Results

Participants’ own experiences with prenatal testing for Down syndrome varied widely as did their attitudes towards prenatal screening and NIPT. Participants discussed four main themes: NIPT test characteristics; consequences of a lower barrier for prenatal screening; requirements for a responsible NIPT offer; and widening the scope of prenatal screening. The findings are summarized below.

### Theme 1: NIPT Test Characteristics: Accuracy, Safety, Earlier Testing

Although not all participants necessarily agreed with prenatal screening, their first impressions of NIPT were positive. Different advantages of NIPT related to its test characteristics were discussed.

#### Accuracy and Safety

The high accuracy of NIPT was seen as an advantage, as participants felt that the test currently used for prenatal screening, the FCT, had limited accuracy, causing unnecessary invasive tests and a false sense of security in women with a low-risk estimation.

*“I had a chance of 1 in 800 [after FCT], well, I had some friends who had a chance of 1 in 20. They did not have a child with Down syndrome, and I did. I was totally not prepared for it, because I actually thought that my child would not have Down syndrome, because I had excluded that with the test [FCT].” (I11)*

Participants stated that NIPT’s ability to reduce the number of invasive procedures, and thus miscarriages, is a great advantage since these tests are risky and stressful, both for pregnant women and obstetricians.

*“Lower risk of miscarriages, and that is of course, the big advantage I think […] I have had chorionic villus sampling, but that’s just not nice. It was a very bad experience […] it was painful but also emotionally a bad experience.” (FG1)*

Most participants argued that because NIPT is accurate and safe, it is easier for women to test whether the fetus has Down syndrome. In the case of a positive test result, this allows women to prepare themselves emotionally for the birth of a child with special needs, arrange adapted perinatal care, or terminate the pregnancy if they feel they are not able to cope with a child with Down syndrome.

*“For me that is the biggest advantage, that without the risk of a miscarriage you know what the situation is and from there on can think: What do I choose?” (FG2)*

*“If NIPT had been available back then, I would have liked to have had it, because then at least I would have known [that the fetus had Down syndrome]. Our child had a very narrow escape [at birth]; there would have been less risk if we had known.” (FG3)*

#### Testing Earlier

The fact that NIPT can test earlier in pregnancy than the FCT was seen as an advantage because participants expected less maternal-fetal bonding during the early phase of pregnancy. Should the fetus have an abnormality, and prospective parents wish to terminate, it was thought that this would be easier to deal with because they are less attached to the child.

*“Yeah I think the earlier you know, the less difficult an abortion will be probably, for me I think, because a child develops so quickly […] I think I would be able to live with it [termination of pregnancy] better if it‘s done as early as possible.” (FG2)*

Participants also mentioned that testing early in pregnancy is better because fewer people are aware of the pregnancy, which means that a potential termination of pregnancy would be easier for the parents socially as they would not have to explain it to others.

While most agreed that earlier testing is an advantage of NIPT, some argued that this could also be a disadvantage. They thought that women (and their partner) would terminate the pregnancy less thoughtfully since they are less involved in the pregnancy at this stage, feel less of a bond with the child, or do not have enough time to think carefully about what they want. They expected this could even lead to regret afterwards.

*“You are maybe less involved with your pregnancy. […] you have thought less well about the consequences of aborting it, while later on you may feel sorry about it.” (FG1)*

### Theme 2: Consequences of a Lower Barrier for Prenatal Screening

Most participants felt that because of the better test characteristics, NIPT would lower the barrier for participation in prenatal screening. Some saw this as an advantage since prenatal screening will become easier as only a blood sample is required, and there is no risk of miscarriage.

*“It is just more accessible because of the fact that there is less risk of a miscarriage […] you can just give blood, so in that sense it is more accessible […] it lowers the barrier.” (FG1)*

Most participants, however, saw this lessened barrier as a disadvantage. Since NIPT is such an easy and risk-free test, it might become more “normalized” to screen for Down syndrome, and Down syndrome would become less accepted.

*“It will become more normal to test for Down syndrome with the consequence that Down syndrome becomes even more undesirable, because the fact that you screen for something means it is undesirable, otherwise you wouldn’t screen for it.” (FG1)*

Moreover, participants thought that pregnant women and their partner might feel pressured by society to have NIPT. Participants stated that already with the FCT some parents of children with Down had the experience of being judged on their choice not to screen. Since NIPT is a better test, women who decline NIPT might feel the need to explain their decision. Having a child with Down syndrome might be regarded as their own responsibility for which society would then be justified to hold them (financially) accountable.

*“Your freedom of choice will be limited in such a way that you have to explain the fact that you don’t want to screen.” (FG3)*

*“Like, you consciously decided not to test, so it’s kind of your own fault…so then you also will carry the burden of it. So everything it [having a child with Down syndrome] costs, yeah: Sorry madam, you should been tested then.” (FG3)*

**Fig. 1:**
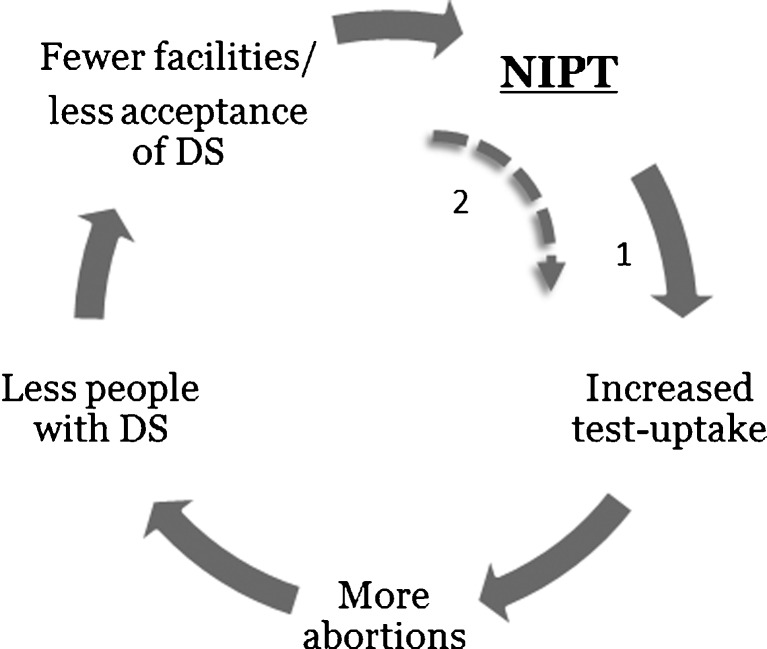
Self-reinforcing process of impact of NIPT, based on expectations of Dutch parents of children with Down syndrome (DS)

Participants thought that the uptake of prenatal screening would increase with NIPT, and more people would terminate their pregnancy. This would cause a decline of the population with Down syndrome, leading to a potential loss of acceptance and facilities for affected individuals. They also were concerned that because of the decreasing number of people with Down syndrome there would be less research on Down syndrome-related complications, thereby eroding the knowledge concerning treatments and care for people with Down syndrome. In this scenario women would not really have a choice anymore to turn down prenatal screening, which would lead to an even higher uptake. This supposed self-reinforcing process is illustrated in Fig. 1.

*“The moment you make screening more accessible and lower the barriers [...] more people will do it […], and as a consequence of that, the population [of people with Down syndrome] will decline. I am sure of that.” (FG1)*

*“What has been fought for, for so long, that those people finally, yeah, are more accepted in society, […] that will all go, well, it might deteriorate.” (FG2)*

Participants hypothesized that the advantages of NIPT are mostly applicable to the individual woman. In contrast, the disadvantages of NIPT are more likely to affect society as a whole. For example, they feared it would lead to a loss of diversity in society. They thought that people with Down syndrome were valuable to society, and that people could learn from them.

*“The way he [son] has contact with other people, everybody can take it as an example. […] he gives a lot of joy, and it sometimes brings you back to reality.” (I3)*

Participants indicated that having a disability could become less acceptable by society.

*“[Screening] affects people with a disability.[…] There is a negative attitude towards people with a disability, and this is stimulated [by the introduction of NIPT].”(I2)*

In addition, participants thought that people may get the idea that life can be controlled by using NIPT, and that this might lead to unrealistic expectations about having children.

*“It’s not like: Okay, I did the test and I am done now, and everything will be fine. Having a child is not easy, and a lot of things can be wrong with the child, and there are external factors that influence child development. Now [with NIPT] it seems like, well you can exclude everything […]. That’s just not true.” (I7)*

Lastly, participants felt that prenatal screening puts a lot of focus on Down syndrome, while trisomy 13 and 18 can also be identified. They felt that with NIPT, the focus is even more on Down syndrome. They stated that in the (Dutch) media NIPT is being called “the Down-test” (e.g. van Calmthout 2013), which in their opinion suggests that Down syndrome is the worst thing that can happen to your child.

*“It [prenatal screening] makes it seem as if the most important thing is to avoid having Down syndrome […] like, when it [the child] has Down syndrome then your world will fall apart, there is nothing worse than that […]. I am not saying it is not a handicap. But it is not the worst in the world, no.” (FG2)*

*“Actually it’s already becoming standard: NIPT equals Down syndrome, which equals terminating the pregnancy.” (FG3)*

Some participants wondered why Down syndrome is still screened for at all. They felt that people with Down syndrome can have a valuable life, and that there has been significant medical progress, giving children with Down syndrome much fewer medical problems nowadays.

*“I support screening if there is something one can do, and if suffering can be avoided. We therefore did the FCT because trisomy 13 or 18…we wouldn’t wish that on a child. But a child with Down syndrome […] overall can have a valuable life in society.” (FG3)*

*“I often wonder for what medical reason they screen for Down syndrome [..] the reason why those children did not survive was primarily because of their heart disorder, and there has been so much medical progress on that.” (FG1)*

### Theme 3: Requirements for a Responsible NIPT Offer

#### NIPT in Public Healthcare

Although not all participants agreed with screening for Down syndrome, most did think it is unrealistic to stop offering prenatal screening. Therefore, when prenatal screening is being offered anyway, they felt that it would be better to screen with a safe and accurate test like NIPT and to embed this in public healthcare with proper counseling, instead of women going to a commercial setting where they might receive poor counseling and information.

*“You’re better off starting to offer it [NIPT] within public healthcare and making sure there is proper counseling than having it offered anyway in some kind of commercial setting.” (FG1)*

#### Reimbursement of NIPT

Participants had trouble deciding whether NIPT should be reimbursed. They felt that by doing so, you send out a certain message that would encourage all people to do this test without thoroughly thinking about it. However, the present cost of NIPT could create double stigmatization, where children with Down syndrome are only born in lower social economic classes, because those people cannot afford NIPT.

*“People with a low income, yeah, they cannot do it [NIPT]. Yeah, it will be like when you could recognize someone’s poverty by the state of his teeth.” (FG3)*

#### Information and Counseling

Almost all participants mentioned that improving information provision during the implementation of NIPT is important to support informed decision-making and avoid routinization. Participants felt improvement to be necessary because they thought there was a lack of good counseling and up-to-date, balanced information about Down syndrome. They also felt that in society Down syndrome is portrayed as being either too negative or too optimistic.

*“We are programmed to think it is terrible to have a child with Down syndrome. But if you see how normal a child with Down syndrome can be, if you, in some way, can incorporate that [in counseling], then you get more balanced information than there is now.” (FG1)*

*“There is a group that portrays it [Down syndrome] as very positive, but they want to counterbalance all those negative stories […] I would like to see a midway, the reality […] just show how it really is, and that is very diverse.” (FG1)*

Several participants mentioned that the government contributes to the negative image of Down syndrome by providing the possibility to test for Down syndrome.

*“Down syndrome really gets labelled as a disorder that should not exist. At least, that’s how many parents [of children with Down syndrome] perceive it, and for that we blame the government.” (I9)*

They indicated that the government therefore has the responsibility to correct the negative image of Down syndrome by, for example, information campaigns, and that the government should not spend money on the implementation of a new test without improving the information provision.

To achieve balanced and complete counseling for NIPT, many parents stated that, in addition to medical information, more information about living with Down syndrome should be given.

*“Yes, also the counseling, […] I think that obstetricians and midwives can still learn a lesson about that when NIPT gets implemented. […] I think counseling is very important. To portray a realistic picture [of] what it’s like to live with a child that has a disorder.” (I10)*

*“I think you should highlight all sides [of Down syndrome]. The current counseling for Down syndrome is like ‘high risk of heart disease,’ ‘higher risk for this’ […] you are just getting a list of symptoms. […] When you offer it [NIPT] to people, you should also offer all information […], all sides of it. Make sure that people really get an honest picture.” (I6)*

Several participants mentioned that parents of children with Down syndrome could have a role as an information source. They could share their experiences of having a child with Down syndrome and make people understand what it is like.

*“Not to convince them [prospective parents], but to tell the truth, to show the reality.” (I8)*

Some participants also thought there was unfamiliarity with Down syndrome amongst healthcare professionals. They were concerned because professionals play a major role during counseling and can have a significant impact on parents’ decisions, as parents might feel uncertain and anxious after receiving test results. Some participants also mentioned that for some obstetricians, a termination of pregnancy is the obvious next step after a Down syndrome diagnosis. Participants therefore stressed the importance of a non-directive attitude of the health professional.

*“That people hear like ‘Well you had amniocentesis, you carry a child with Down syndrome, so when are we going to set the appointment to terminate the pregnancy?’” (FG1)*

*“I can imagine that, when you are pregnant and have a lot of hormones and emotions and whatever, and then you hear that your child has Down syndrome and you know nothing about it, then you get the opinion of a doctor. The question is whether all doctors will have the same opinion. I think not.” (I1)*

### Theme 4: Widening the Scope of Prenatal Screening with NIPT

Participants had conflicting thoughts about testing for more disorders with NIPT. They agreed it had a number of advantages, like being able to prevent suffering, to arrange adapted perinatal care, or starting soon after birth with a certain diet to lessen the pathology of the disorder.

*“If people indeed happen to have a disorder that you can, for example, partly prevent with a lifestyle or diet […] yeah that of course has its advantages.” (FG1)*

Some participants mentioned that it would give parents the option to decide whether they would be capable of caring for a child with a disorder.

*“I find the freedom of choice of parents very important. Like, can I handle this? Will we be able to deal with this in my family?” (FG2)*

Some participants also felt that testing for more disorders could lessen the focus on Down syndrome, which they saw as a benefit.

*Moderator: “Expanding the offer [of NIPT] to other disorders, what do you think about that?”*

*Respondent: “Well, I think, that as long as it [a broader NIPT test] goes along with good information provision...look, what I find wrong at this moment is that the focus is so much on Down syndrome […] and if there will be more [disorders], […] as long as the information provision is right, everyone should be able to decide for themselves.” (FG1)*

Participants expected it to be difficult to decide where to draw the line when testing for a broader range of disorders, and to avoid that this line getting crossed over time.

*“Yeah, what would worry me a lot is how to guard that line […] what we can all test for. We are curious by nature you know, there will always be people that will want to cross that line.” (FG1)*

Some participants noted that it is not up to prospective parents to decide about everything since we cannot control everything in life. Some also mentioned that society would not benefit from eliminating everything that differs from the “normal standard.”

*“I find it very dangerous that as a society we more and more make value judgments on everyone who doesn’t fit the strict definition of normality.” (FG3)*

Other participants mentioned that people would be faced with even more difficult decisions to deal with during pregnancy. Moreover, they worried what kind of impact it would have on eligibility for healthcare insurance or housing mortgages.

## Discussion

Parents of children with Down syndrome considered the accuracy, safety and possibility to test earlier as advantages of using NIPT in prenatal screening. However, they thought that prenatal screening in general, and the use of NIPT in particular, put too much focus on Down syndrome, making it seem like Down syndrome is the worst thing that can happen to one’s child. They expected that NIPT would lower the barrier for participation in screening, which has both advantages and disadvantages. Participants argued that NIPT gives people a more accurate option to test for Down syndrome without having to risk a miscarriage; but because of that, testing for Down syndrome and terminating the pregnancy could also become more normal. They feared the latter could erode the acceptance, facilities and research for Down syndrome, which in turn leaves women with little room to decline testing (self-reinforcing process illustrated in Fig. 1). Participants stated that, when implementing NIPT, the counseling should be improved by giving more balanced, accurate information, including more information about living with Down syndrome. Although participants assumed that testing for more disorders with NIPT diverts the focus away from Down syndrome and allows for early medical intervention, they worried about where to draw the line. They also feared a loss of diversity in society.

This study describes the views of a sample of parents and relatives of children with Down syndrome in the Netherlands, a country with relatively low uptake of prenatal screening. When compared with the attitudes of pregnant women in the Netherlands as well as pregnant women in other countries (Farrell et al. 2014; Lewis et al. 2013; van Schendel et al. 2014), it seems that parents of children with Down syndrome often perceive similar advantages and disadvantages of NIPT. Like pregnant women, they believe NIPT lessens the barrier for participation in screening because it is a simple and safe test that can be done early in pregnancy. Similar to the study by Kellogg et al. (2014) of mothers of children with Down, participants agreed the lower barrier is beneficial because it allows people to test without risk and decide, after confirmation, whether or not to continue the pregnancy based on that information.

The notion that it could also lead to an increase in termination of pregnancies also corresponds to findings of Kellogg et al. (2014), where the majority believed NIPT would lead to the termination of more pregnancies. A study by Natoli et al. (2012) on termination rates after a Down syndrome diagnosis showed that higher termination rates were associated with earlier gestational age. This finding supports the assumption that NIPT’s ability to test earlier could lead to more termination of pregnancies, although others have suggested that with NIPT the percentage of women who opt for termination of pregnancy in the case of an affected pregnancy may decrease (Verweij et al. 2013).

The fear expressed by participants that fewer children with Down syndrome being born could lead to stigmatization and fewer facilities, is a concern that was also observed in several other studies (Allyse et al. 2015; Haider et al. 2016; Lewis et al. 2013; van Schendel et al. 2014). Due to the lower barrier for NIPT, participants indicated that good quality counseling and informed decision-making are of great importance. This awareness also exists amongst health professionals, for example, genetic counselors from the UK, who stated that because NIPT has the potential to become routinized, it is the professional’s role to make sure that women understand what they are consenting to (Alexander et al. 2014).

To help healthcare professionals facilitate meaningful discussions between themselves and prospective parents, Sachs et al. (2015) have developed a framework for pre-test counseling about NIPT, especially focusing on its capabilities and limitations. Participants in our study, however, felt that already in current screening practice, information and counseling were not up to standard. They were especially critical of the quality of the information about Down syndrome given at different stages of the screening trajectory. Studies in other countries suggest that knowledge of Down syndrome among healthcare professionals could be improved (Ternby et al. 2015a), and that some parents perceive the information about what it may mean to live with this condition, both for the individual and for the parents, as insufficient (Carroll et al. 2012; Ternby et al. 2015b) or overly negative (Kellogg et al. 2014). It was also noted that the information leaflets for those considering screening for Down syndrome should provide more accurate information about this (Saiklang and Skirton 2015). Participants in our study thought that parents of children with Down syndrome could play a valuable role in this respect as well.

Similar to pregnant women (van Schendel et al. 2014), parents of children with Down syndrome think that testing for more disorders with NIPT can have some advantages. Interestingly, one of the advantages mentioned was that it would shift the focus away from Down syndrome, thus avoiding the impression of Down syndrome as a disorder for which screening would somehow be more justified than for other (including more serious) conditions, something that many of these parents find unjust and hurtful. Participants, however, feared testing for more disorders would confront prospective parents with even more difficult decisions. This fear was also expressed in our previous questionnaire study of Dutch pregnant women, who stated that “testing for a broad range of disorders may complicate the decision-making process beyond what most couples are able to comprehend” (van Schendel et al. 2015). Although it was not explicitly mentioned in this study, widening the scope of testing will also make it increasingly difficult to meaningfully discuss prior to testing what it is like to have a child with any of the conditions screened for. Participants in our study also feared a loss of diversity in society, which is in line with findings from a previous study in the UK that highlighted public fears of fueling a problematic quest for perfection if NIPT were to be used to screen for an ever wider range of disorders (Kelly and Farrimond 2012).

### Study Limitations and Research Recommendations

A strength of this study is the qualitative approach, which allows for exploring in-depth views about NIPT. Using both focus groups and individual interviews allowed us to explore opinions in both a group context and more private environments, which strengthened the credibility of the results. As far as we know, this is the first qualitative study of the attitudes of parents of Down syndrome children towards NIPT. A limitation of the study is that almost all participants were recruited from one source, a Facebook group which consisted of people with relatively young children. Moreover, participants were Caucasian and highly educated. This might have led to biased responses. Additionally, previous discussions on this Facebook page might have influenced participants’ opinions. Moreover, attitudes of parents might have been influenced by the strongly articulated opinions in the Dutch media. In the focus group and individual interviews held in 2015, participants seemed more negative about NIPT than in the focus groups in 2013. However, the sample size is too small to draw conclusions on this point. The study was conducted in the Netherlands, where prenatal testing is offered in a nationally organized prenatal screening system, the uptake of which is relatively low. Attitudes of participants in this study may thus vary from those living in countries where NIPT is offered by individual (commercial) healthcare providers, or in countries with other cultures and religions. Finally, qualitative data are not intended to be generalized to the population of interest. Future studies might include larger samples of males and females. Moreover, should NIPT be introduced as a first-tier screening test, it would be interesting to see whether and how it affects parents’ opinions.

### Conclusion and Practice Implications

The findings from this study provide insight into the expectations and concerns that parents of children with Down syndrome have about introducing NIPT into a national prenatal screening system. It can be concluded that parents of children with Down syndrome may have ambivalent attitudes towards NIPT. While they do not necessarily all agree with prenatal screening, they do acknowledge that NIPT offers a better option than the combined test to know whether the fetus has Down syndrome. However, they also expressed concerns for the future of children with Down syndrome and emphasized the need for good counseling and information provision, including more information about living with Down syndrome.
